# Benign gastro-bronchial fistula – an uncommon complication of esophagectomy: case report

**DOI:** 10.1186/1471-2482-5-16

**Published:** 2005-06-30

**Authors:** Mohan P Devbhandari, Rohit Jain, Simon Galloway, Piotr Krysiak

**Affiliations:** 1 Department of Cardiothoracic surgery South Manchester University Hospital, NHS Trust, Southmoor Road, Wythenshawe, Manchester, M23 9LT, UK; 2 Department of General Surgery South Manchester University Hospital, NHS Trust, Southmoor Road, Wythenshawe, Manchester, M23 9LT, UK

## Abstract

**Background:**

Gastro-bronchial fistula (GBF) is a rare and devastating complication following esophagectomy. Making the correct diagnosis is difficult and there is no agreement on the treatment for this rare condition.

**Case presentation:**

We report the case of a 56-year-old man who presented with features of repeated aspiration and chest infections six years following an esophagectomy for Barrett's esophagus. Despite extensive investigations the cause of symptoms was difficult to determine. The correct diagnosis of fistula from stomach to right main stem bronchus was made at bronchoscopy under general anesthesia. After ruling out local recurrence of cancer, a successful primary repair was carried out by resection of fistula and direct repair of gastric conduit and bronchus. He is well after 6 months of treatment.

**Conclusion:**

Late development of gastro-bronchial fistula is a rare complication of esophageal resection that may be difficult to diagnose.

Surgical resection and direct closure is the treatment of choice, although the method of treatment should be tailored according to the anatomy of the fistula and the patient's condition.

## Background

Gastrobronchial fistula (GBF) following esophagectomy for malignant disease is a recognized complication. However benign GBF are rare and the literature on this complication is limited consisting of a handful of case reports. Their mode of presentation and management are varied and there is no single treatment method, which is widely accepted. We report a case, which was successfully treated by direct primary repair. A brief review of literature is presented.

## Case presentation

A 67-year-old man with past history of Ivor Lewis esophagectomy 6 years previously for a Barrett's esophagus presented with dysphagia, recurrent cough, regurgitation and weight-loss. Endoscopy suggested a benign anastomotic stricture, for which he underwent dilatation. However his symptoms worsened and he continued to lose weight in spite of supervised dietary therapy. Aspiration was suspected and a barium swallow was performed. This demonstrated pharyngeal in-coordination, as the probable cause of aspiration. A repeat endoscopy was non-contributory. A feeding jejunostomy was inserted to prevent continued weight loss. In spite of this, he continued to have repeated chest infections resulting in extensive bronchiectatic changes in the right lower lobe.

A tracheostomy was planned at this stage to prevent aspiration. Whilst he was being intubated for the procedure, air was noted to escape from the right bronchus. A bronchoscopy revealed the presence of the GBF, with the bronchial opening in the right main stem bronchus located about 1 cm below the tracheal bifurcation and the gastric opening midway down the lesser curvature of the gastric conduit. The temporal sequence of events suggests that fistulation had occurred at the time of anastomotic dilatation, and thus the worsening of symptoms following the procedure. The fistula was however, difficult to diagnose in spite of extensive investigations leading to unexpected finding in the anaesthetic room.

**Figure 1:**
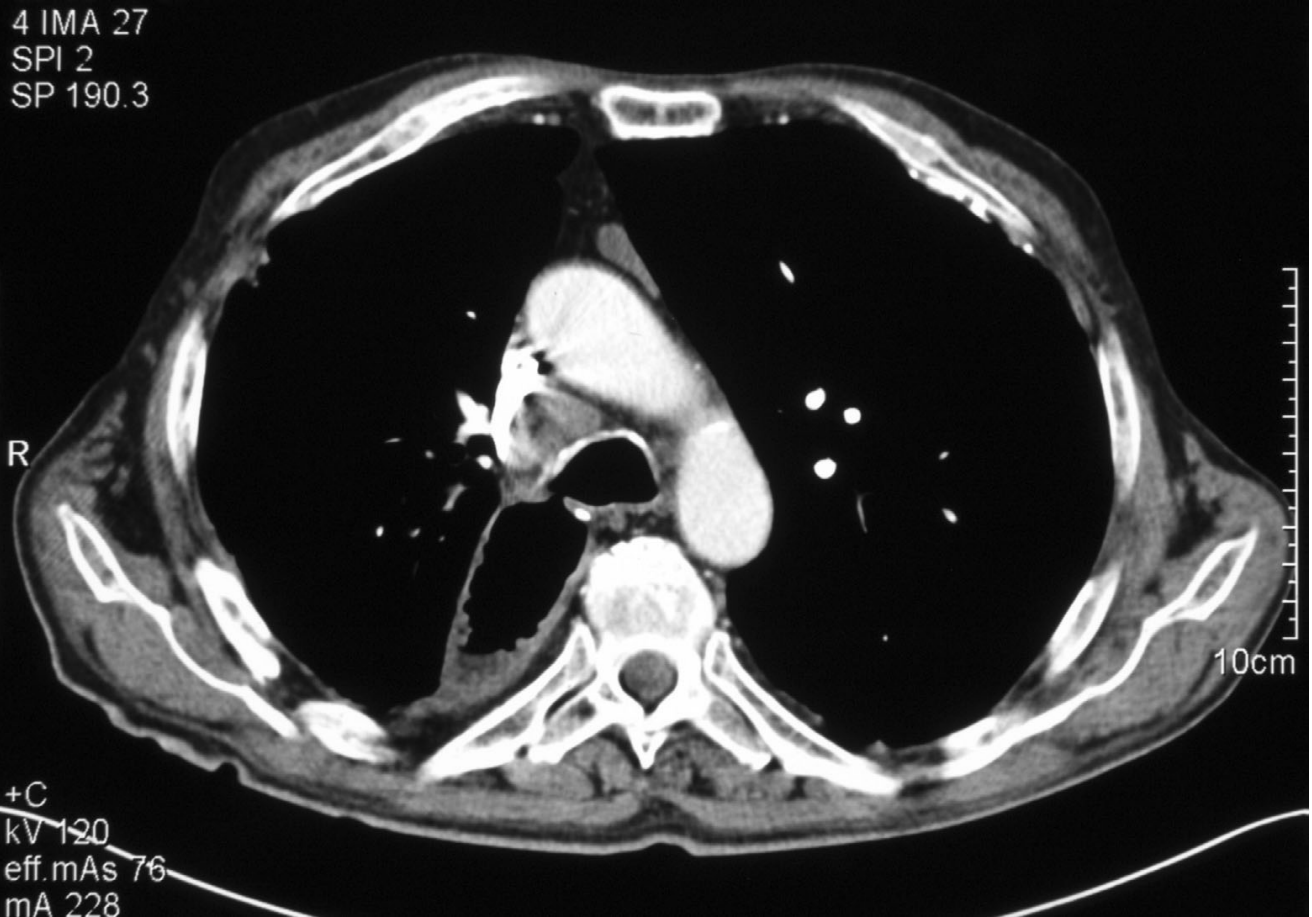
CT scan of chest of showing communication between the right main bronchus and the gastric conduit.

Given his debilitated condition and poor respiratory function stenting was considered to occlude the fistula. However as the fistula was positioned in the middle of the gastric conduit (Figure 1) it was not deemed amenable to stenting due to poor fixation of the stent and risk of migration. And similarly satisfactory stenting of right main stem bronchus was difficult to achieve due to short length of this bronchus and consequent tendency to migrate. Though associated with greater risk of morbidity and mortality, a surgical approach was deemed the best approach for this problem.

**Figure 2:**
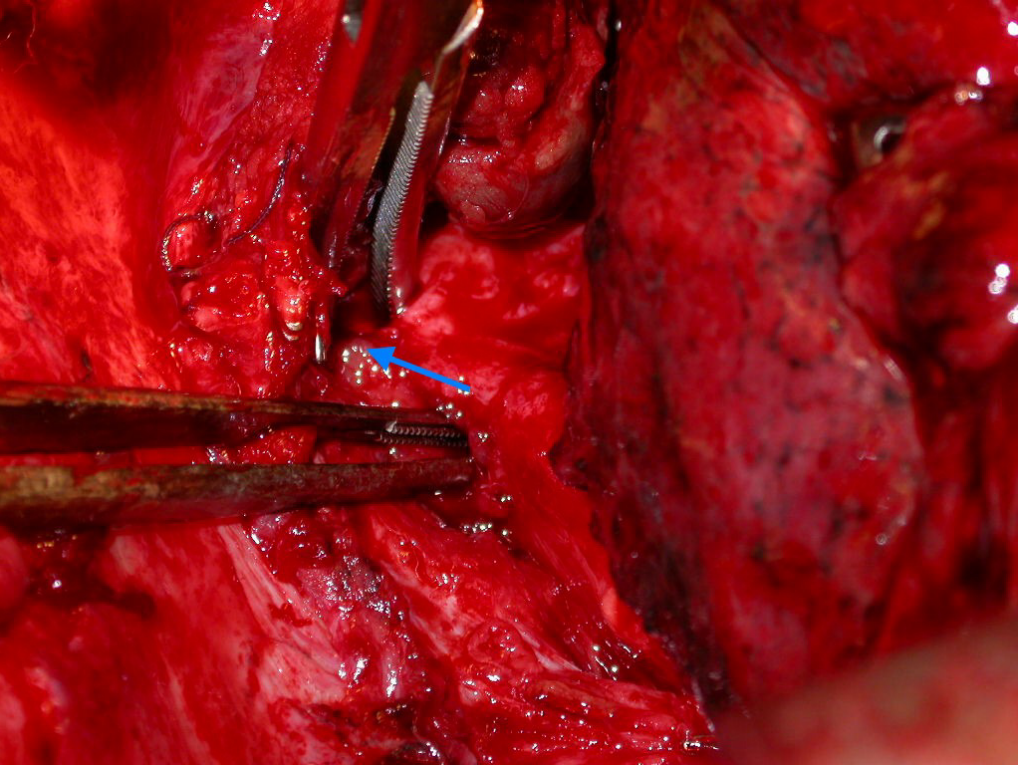
Intra-operative picture showing the fistula (arrowed) between two forceps.

At operation a right-sided re-thoracotomy was performed and careful dissection carried out to display the anatomy of the GBF (Figure 2). The fistula was excised with a cuff of healthy gastric conduit and bronchial tissue. The gastric and bronchial openings were closed directly with absorbable stitches with interposition of bovine pericardium in between the two suture lines. The patient made an uneventful recovery from operation. A contrast study one-week later confirmed absence of leakage from the gastric conduit and feeding was started. Histopathological examination confirmed the presence of benign GBF in the resected specimen.

The patient was discharged home after two weeks and remains well six months later.

## Discussion

GBF is a rare complication in patients with an intra-thoracic oesophagogastric anastomosis after esophageal resection. Recurrent malignancy is the commonest cause of this complication. Non-malignant cases may occur early or late. In the early postoperative period GBF usually arises as a result of the extensive dissection, ischemia [1] or surgical staples [2]. It may however present late as this case, several years after the operation. In such cases the causes are chronic peptic ulcer in the gastric conduit, traumatic anastomotic dilatation [3,4] or infection.

The usual modes of presentation are cough associated with eating, dyspnoea, haemoptysis and recurrent chest infections. The fistula may connect at any site in the respiratory tract, from trachea down to lobar bronchus. The fistula may be difficult to locate and may not be visualized at endoscopy of respiratory or upper GI tract. In such cases a methylene blue dye test may show bluish sputum when the patient is asked to swallow the dye. CT scan or upper GI contrast radiology is also helpful in arriving at the correct diagnosis. Multiple biopsies help to rule out recurrence of malignancy as the cause. Left untreated, GBF is usually fatal due to chronic pulmonary sepsis and one should not rely on conservative treatment [5].

Patients often present in poor general condition with malnutrition and chronic pulmonary infection. Prompt institution of broad-spectrum antibiotic cover, gastric drainage, attention to fluid and electrolyte balance, nutritional support and chest physiotherapy are essential steps in preparation for definitive surgery. In acute cases with the presence of gross mediastinal sepsis cervical oesophagostomy and return of the stomach tube to abdomen with débridement is performed. The continuity can be restored at a later date with an alternative conduit e.g. colonic interposition.

The ideal operation consists of re-thoracotomy and resection of fistula with direct closure of the openings in the esophagus and the respiratory tree, preferably with an intervening viable tissue. A variety of tissue and pedicles of muscles like pectoralis major, intercostal, latissimus dorsi [4] and sternocleidomastoid [3] have been used to interpose between the two repaired tubes. Where required the membranous portion of airway can be substituted with fascia lata, autologous pericardium or bovine pericardium to close the defect. A defect in the lower lobe bronchus is most often managed by lower lobectomy where as a defect in the main-stem bronchus may be resected and repaired [5]. GBF arising around the level of the oesophagogastric anastomosis may not be amenable to resection and direct closure. A tension free well-vascularized closure is crucial in the success of this procedure and an alternative conduit may be required. No one single procedure is suitable for all the patients and the surgeon should be aware of the options available.

Late development of GBF is a rare complication of esophageal surgery that may be difficult to diagnose. Surgical resection and direct closure is the treatment of choice. However where patients are debilitated or if there is insufficient length of airway available for resectional surgery, alternative, less invasive endoscopic stenting procedures may be used [6].

## Conclusion

Late development of gastro-bronchial fistula is a rare complication of esophageal resection that may be difficult to diagnose.

Surgical resection and direct closure is the treatment of choice, although the method of treatment should be tailored according to the anatomy of the fistula and the patient's condition.

## List of abbreviations used

GBF : Gastrobronchial Fistula

## Competing interests

The author(s) declare that they have no competing interests.

## Authors' contributions

**MPD **collated the information, searched literature and wrote the manuscript.

**RJ **assisted in literature search, writing of the manuscript, editing the final script and doing the final submission.

**SG **and **PK **assisted in providing a critical appraisal and review of the manuscript.

## Pre-publication history

The pre-publication history for this paper can be accessed here:



## References

[B1] Kalamar  K, Molnar TF, Horvath OP (1999). Two cases of benign tracheo-gastric fistula following esophagectomy for cancer. Acta Chir Hung.

[B2] Pramesh CS, Sharma S, Saklani AP, Sanghvi BV (2001). Broncho-gastric fistula complicating transthoracic esophagectomy. Dis Esophagus.

[B3] Sakamoto K, Ogawa M, Yamamoto S, Mugita N, Saishoji T, Azuma AS, Hayashida K (1997). Closure of gastric tube-tracheal fistula by transposition of a pedicled sternocleidomastoid muscle flap. Surg today.

[B4] Aguilo Espases R, Lozano R, Navarro AC, Regueiro F, Tejero E, Salinas JC (2004). Gastrobronchial fistula and anastomotic esophagogastric stenosis after esophagectomy for esophageal carcinoma. J Thorac Cardiovasc Surg.

[B5] Brega Massone PP, Infante M, Valente M, Conti B, Carboni U, Cataldo I (2002). Gastrobronchial fistula repair followed by esophageal leak – rescue by transesophageal drainage of the pleural cavity. Thorac Cardiovasc Surg.

[B6] Bennie MJ, Sabharwal T, Dussek J, Adam A (2003). Bronchogastric fistula successfully treated with the insertion of a covered bronchial stent. Eur Radiol.

